# The effects of resting and exercise serum from children with cystic fibrosis on C2C12 myoblast proliferation in vitro

**DOI:** 10.14814/phy2.12042

**Published:** 2014-06-24

**Authors:** Thanh Nguyen, Jeff M. Baker, Joyce Obeid, Sandeep Raha, Gianni Parise, Linda Pedder, Brian W. Timmons

**Affiliations:** 1 Child Health & Exercise Medicine Program, McMaster University, Hamilton, Ontario, Canada; 2 Exercise Metabolism Research Group, Department of Kinesiology, McMaster University, Hamilton, Ontario, Canada; 3 Department of Pediatrics, McMaster University, Hamilton, Ontario, Canada; 4 Cystic Fibrosis Clinic, McMaster Children's Hospital, Hamilton, Ontario, Canada

**Keywords:** C2C12 cells, children, exercise

## Abstract

Chronic systemic inflammation is a clinical symptom in children with cystic fibrosis (CF), but the effects on skeletal muscle development are unknown. The aims of this study were to determine (1) the effects of systemic factors from children with CF and healthy controls on myoblast proliferation, and (2) whether exercise serum can have an effect on proliferation in vitro. Eleven children with CF and 11 biological age‐matched controls completed two 30‐min bouts of cycling at an intensity set at 50% peak mechanical power. Serum samples were collected before exercise (REST), immediately following exercise (EX), and after 60 min of recovery (REC). Serum samples prepared in group‐specific pools were used for cell culture experiments. C2C12 myoblasts were incubated in 5% serum and media for 1 h and then immediately harvested for protein and mRNA analysis, or incubated in growth media for 2 days to examine proliferation. C2C12 myoblasts treated with CF serum displayed greater proliferation phenotype than myoblasts treated with control serum. Proliferation did not change with EX or REC serum from children with CF compared to CF REST serum, while proliferation was increased with EX and REC serum from control compared to control REST serum. These findings suggest that systemic factors from children with CF at rest and after exercise can alter myoblast proliferation responses when compared to systemic factors from healthy children, which may have implications on skeletal muscle development.

## Abstract (precis)

Chronic systemic inflammation is a clinical symptom in children with cystic fibrosis (CF), but the effects on muscle development are unknown. We exposed C2C12 myoblasts to the serum of children with CF and healthy children, and found that systemic factors from children with CF at rest and after exercise can alter myoblast proliferation.

## Introduction

Children with cystic fibrosis (CF) suffer from chronic systemic inflammation (Tirakitsoontorn et al. 2001; Nguyen et al. 2012) that may negatively impact whole body skeletal muscle mass via increased protein catabolism (van Heeckeren et al. 2000; Ionescu et al. 2002, 2004). In patients with CF, those who have increased protein breakdown as measured by urinary pseudouridine, tend to have increased IL‐6 and TNF‐*α* (Ionescu et al. 2002), and lower fat‐free mass (Ionescu et al. 2002, 2004). This systemic inflammation observed in patients with CF may stem from chronic pulmonary infection (Valletta et al. 1992; van Heeckeren et al. 2000). Indeed, mice infected with *Pseudomonas aeruginosa* localized in the lungs have increased pulmonary inflammation in the days following infection. This was mirrored by an increase in the inflammatory cytokine, IL‐6, whereby the concentration of IL‐6 observed in the lungs correlated with the amount of weight loss observed in these infected mice, with evidence of reduced skeletal muscle leg mass (van Heeckeren et al. 2000). Collectively, the available evidence suggests that factors in the systemic circulation may negatively affect whole body skeletal muscle development. However, few studies have addressed this important issue in children with CF by studying the specific effects of the systemic environment on molecular and signaling pathways of skeletal muscle development.

Systemic inflammatory profiles can be transiently altered with acute bouts of exercise, even in children with CF (Nguyen et al. 2012). Often an increase in inflammatory cytokines is observed with exercise; however, an exercise‐induced increase in IL‐6, for example, is viewed by many as being anti‐inflammatory (Petersen and Pedersen 2005). Given that chronic levels of systemic inflammation may have a negative impact on whole body skeletal muscle, but that specific episodes of exercise may create an anti‐inflammatory systemic environment, we wanted to examine the effects of these different systemic environments on indices of skeletal muscle development. Specifically, we wanted to compare the effects of serum from healthy children with serum from children with CF, in rest, exercise, and recovery conditions, on markers of skeletal muscle development.

Since skeletal muscle from children is difficult to obtain for ethical reasons, we used the well‐established C2C12 skeletal muscle murine cell line as the target cells to examine the effects of systemic environments on indices of muscle development. C2C12 cells are a subclone of myoblasts from muscle in the leg of a C3H mouse (Yaffe and Saxel 1977; Burattini et al. 2004) and are responsive to human factors (Sadowski et al. 2001; Frost et al. 2002). Muscle development encompasses several stages, including proliferation, differentiation, and fusion. We examined aspects of myoblast proliferation as it represents the early stages of muscle development (Peault et al. 2007). Specifically, we determined how different systemic environments affected proliferation signaling pathways and gene expression, and whether these molecular changes translated into an altered phenotype of proliferation, as measured by an increased number of cells. To provide additional insight into the regulation of skeletal muscle development, we also examined markers of differentiation. Since inflammation can induce greater C2C12 myoblasts proliferation at the cost of reduced differentiation into myotubes (Dogra et al. 2006), we hypothesized that compared with healthy controls, serum from children with CF would induce greater signaling of proliferation pathways due to their chronic systemic inflammation (Nguyen et al. 2012), resulting in a greater proliferation phenotype. Moreover, given that IL‐6 is associated with increasing myoblast proliferation (Toth et al. 2011) and exercise is known to increase circulating IL‐6 (Nguyen et al. 2012), we hypothesized that changes in the systemic environment induced by a specific episode of exercise would enhance proliferation.

## Methods

### Participants

Participants' characteristics are shown in Table 1. Eleven children with CF (two females) with complete blood samples were included in this study. Patients were recruited from the Cystic Fibrosis Clinic at the McMaster Children's Hospital (Hamilton, Ontario, Canada). Children with CF who could not perform reproducible pulmonary function tests were excluded from the study. Six patients with CF were taking nonsteroidal anti‐inflammatory drugs (NSAIDs) and/or inhaled or nasal spray corticosteroids. One participant was diagnosed with cirrhosis of the liver. Eleven sex‐ and biological age‐matched (Mirwald et al. 2002) healthy control children were recruited from the surrounding community. Healthy control children were included in the study only if they had no known physical, mental, metabolic, and inflammatory diseases. All parents/guardians and children provided written informed consent and assent, respectively, prior to enrollment in this study, which was approved by the Hamilton Health Science/Faculty of Health Science Research Ethics Board, with consent and assent forms signed by parents/guardian and children, respectively.

**Table 1.** Participants' characteristics.

	CF (*n *=**11)	Matched controls (*n *=**11)	*P* value
Age (years)	14.4 ± 2.2	13.9 ± 2.2	0.59
Estimated years from predicted age of PHV	0.56 ± 1.71	0.72 ± 1.57	0.83
FEV_1_ (% predicted)	90.3 ± 22.0	93.4 ± 7.4	0.73
Height (m)	1.62 ± 0.13	1.68 ± 0.13	0.33
Height (‰)	45.5 ± 22.3*	73.7 ± 29.6	0.02
Weight (kg)	49.5 ± 11.9	54.6 ± 9.4	0.27
Weight (‰)	37.2 ± 18.1*	62.2 ± 25.2	0.02
BMI (‰)	35.6 ± 19.4	48.9 ± 23.2	0.16
% Body fat	20.0 ± 4.8	19.2 ± 9.6*	0.79
FFM (kg)	39.8 ± 10.2	44.3 ± 9.7*	0.31

Values are expressed in mean ± SD. PHV, peak height velocity; FEV_1_, forced expiratory volume in 1 sec; ‰, percentile; BMI, body mass index; FFM, fat‐free mass.

* Significant difference between groups.

* *n *=**10 for age‐matched controls.

### Exercise and blood sampling

Participants completed two visits. During the first visit, anthropometric variables (height, weight, body composition using bioelectrical impedance analysis) and FEV_1_ were measured along with peak mechanical power (PMP), assessed using the *McMaster All‐Out Progressive Continuous Cycling Test* on a cycle ergometer (Fleisch‐Metabo, Geneva, Switzerland). Height, weight, and body mass index (BMI) percentiles were calculated using reference values of weight‐for‐age and stature‐for‐age from the Centers for Disease Control and Prevention (2009). Fat‐free mass (FFM) was calculated using an age‐specific BIA equation from Schaefer et al. (1994). Percent body fat was calculated as ([Body weight − FFM]/body weight) × 100. Reference data for FEV_1_ were obtained from Wang et al. (1993) and were used to calculate percent predicted.

The second visit was scheduled a minimum of 2–3 days after the first. Participants were asked to refrain from consuming any food or liquid, with the exception of water, 3 h prior to the visit. They also refrained from participating in any strenuous physical activity for at least 24 h before the visit. The second visit consisted of 2 × 30‐min bouts of cycling at a constant pace of ~60 rpm and an intensity equivalent to 50% PMP. We chose to study the effects of 60 min of moderate‐intensity exercise as this reflects the internationally accepted recommendation for daily physical activity for children (Tremblay et al. 2011; World Health Organization 2013). Blood samples were collected using an in‐dwelling catheter placed in the antecubital region of the arm. Blood samples were collected before exercise (REST), at the end of the 2 × 30‐min bouts of cycling (EX) and after 60 min of recovery (REC). Blood was collected into vacutainers that were either placed on ice (plasma) or allowed to clot for 30 min at room temperature (serum). Samples were then centrifuged for 20 min at 2000*g* and 4°C. All plasma and serum samples were aliquoted and stored at −80°C for future analysis. Plasma samples were analyzed for the inflammatory cytokine IL‐6 and serum samples were used for cell culture experiments.

### Cell culture experiments

To provide a sufficient volume of serum to execute the proposed cell culture experiments, pooling of serum was necessary (i.e., the amount of blood required from one child surpassed what we considered to be ethically justified). Hence, we prepared group‐specific pools of serum (i.e., CF and healthy controls), representing equal volume from each individual within a group, for each of the REST, EX, and REC time points.

### C2C12 myoblasts

C2C12 myoblast cell line was purchased from American Type Culture Collection (Rockville, MD). Myoblasts were grown on 100‐mm cell culture dishes in growth media (GM) consisting of Dulbecco's Modified Eagle's Medium (DMEM) supplemented with 10% fetal bovine serum (FBS) and 1% penicillin–streptomycin and incubated at 37°C in 5% CO_2_.

### Protein and gene expression

Cells at passage 8 were grown until 70% confluence was reached at which point the cells were used in the experimental setup. For treatment, GM was removed and the cells were washed with PBS. Cells were then given 7 mL of treatment consisting of DMEM supplemented with 5% serum from children with CF or from healthy controls, with each time point represented (i.e., REST, EX and REC) and 1% penicillin–streptomycin. Cells were incubated at 37°C in 5% CO_2_ for 1 h, after which treatment media was removed, cells were washed with PBS, and then harvested with 1 mL of TRIzol^®^ Reagent (Life Technologies, Burlington, Ontario, Canada). Plates were placed on ice and samples were collected and aliquoted into 2‐mL tubes. Samples were vortexed and stored at −80°C for further analyses.

### Protein isolation

Samples in TriZol reagent were thawed and treated with 0.2 mL of chloroform, shaken for 15 sec, incubated at room temperature for 5 min, and centrifuged at 12,000*g* and 4°C for 15 min. The upper aqueous phase was placed in 2.0‐mL tubes and stored at −80°C for RNA isolation. The interphase was discarded and the phenol–chloroform phase was placed in a 2.0‐mL tube for protein isolation. Protein isolation was completed using the protein precipitation method as per manufacturer's instruction (TriZol reagent, Life Technologies). Protein pellets were resuspended using 200 *μ*L of 1% SDS with protease inhibitors (Complete mini; Protease inhibitor cocktail tablets; Roche Diagnostics, Laval, Quebec, Canada) and phosphatase inhibitors (PhosSTOP, Roche Diagnostics, Laval, Quebec, Canada). Pellets were incubated at room temperature in resuspension reagents for 20–40 min and then heated at 50°C in a heat block until completely resuspended. Samples were centrifuged at 10,000*g* for 10 min at 4°C and transferred to a new tube. Protein concentration was assessed using Pierce® BCA protein assay kit (Thermo Fisher Scientific Inc., Rockford, IL), and samples stored at −20°C until further analyses.

### Western blotting

We chose to study proteins involved in the JAK/STAT3 pathway, as it has been identified to result in increased C2C12 myoblast proliferation when activated (Spangenburg and Booth 2002). Specifically, STAT3 and its activated form (p‐STAT3) were measured. Suppressor of cytokine signaling (SOCS3) is a key regulator of inflammation and its upregulation inhibits the JAK/STAT3 pathway (Croker et al. 2008; Diao et al. 2009). STAT3, pSTAT3, and SOCS3 protein expressions were analyzed with actin used as a loading control. Equal amounts of protein (5 mg for STAT3; 25 mg for p‐STAT3 and SOCS3) and Laemmli buffer were boiled at 95°C for 5 min. Samples were loaded on in the wells of a 12.5% gel and run at 120 V for approximately 2 h and transferred to polyvinylidene fluoride (PVDF; Millipore, Etobicoke, Canada) membranes at 120 V on ice for 1 h. Membranes were blocked with 5% nonfat powdered milk in 1x TBST at room temperature for 1 h, then incubated overnight in primary antibody (STAT3 rabbit antibody, dilution 1:2000, catalog number #4904. pSTAT3 Tyr705 rabbit antibody, dilution 1:500, catalog number #9145, Cell Signaling Technology, Boston, MA; SOCS3 rabbit antibody, dilution 1:500, catalog number #ab16030, Abcam Inc., Cambridge, MA; Actin rabbit antibody, dilution 1:1000, catalog number # A2066. Sigma‐Aldrich Co., St. Louis, MO) in either 5% BSA (BSA, Santa Cruz Biotechnology, Santa Cruz, CA) in 1x TBST for STAT3, p‐STAT3, SOCS3 or 5% nonfat powdered milk for actin at 4°C. Following multiple washes with 1x TBST, blots were incubated in goat anti‐rabbit HRP (dilution 1:2000, catalog number #7074, Cell Signaling Technology) in 5% nonfat powdered milk in 1x TBST for 60 min at room temperature. Following multiple washes with 1x TBST, proteins were detected with ECL (SuperSignal West Dura, Thermo Fisher Scientific) using FluorChem SP (Alpha Innotech Corporation, San Leandro, CA). Protein bands corresponding to the predicted molecular weight of STAT3 and p‐STAT3 (94 kDa), SOCS3 (27 kDa), and actin (42 kDa) were quantified using the FluorChem SP Software with background correction.

### RNA isolation and reverse transcription

Ribonucleic acid was isolated with E.Z.N.A Total RNA Kit I (Omega Bio‐Tek, Norcross, GA) using the previously isolated upper aqueous phase. Total RNA isolation was carried out using the Omega protocol. RNA was transcribed to 500 ng of cDNA using the Applied Biosciences High Capacity cDNA reverse Transcription Kit (Applied Bioscience, Foster City, CA) and the Eppendorf Mastercycler epigradient thermal cycler (Eppendorf, Mississauga, Ontario, Canada).

### Quantitative real‐time polymerase chain reaction

Quantitative real‐time polymerase chain reaction (qRT‐PCR) was carried out using SYBR Green PCR master mix (AP Applied Biosystems, Warrington, UK) and 25‐*μ*L reactions. Primers were custom made and purchased from the MOBIX Lab (DNA sequencing and Oloigo Synthesis Facility, McMaster University, Hamilton, Canada). Markers of proliferation measured were SOCS3 and Pax7. Pax7 was measured as it is expressed by proliferating myoblasts (Buckingham 2007). In addition to proliferation markers, a differentiation marker was measured to gain additional insight into aspects of muscle development. The differentiation marker measured was myogenin, which is an early marker of differentiation that signifies commitment to myotube development (Andres and Walsh 1996). The primer sequences used are shown in Table 2. GAPDH was used as the housekeeping gene. Primers were reconstituted using 1x TE buffer of pH 8.0 to make 100 *μ*mol/L and stored at −20°C until further analyses. In PCR 0.2‐mL tubes (Axygen Inc., Union City, CA), 12.5 *μ*L of SYBR green, 2 *μ*L forward primer, 2 *μ*L reverse primer, 7.5 *μ*L of H_2_O, and 1 *μ*L (25 ng) of cDNA were combined to give a total volume of 25 *μ*L for all mRNA markers except GAPDH. For GAPDH, 6.5 *μ*L of H_2_O and 2 *μ*L (50 ng) of cDNA was used instead. qRT‐PCR was performed using a Eppendorf Mastercycler ep realplex^2^ real‐time PCR system (Eppendorf). GAPDH expression was not different between conditions. Changes in gene expression over time were expressed as fold change from REST values, using the *∆∆*CT method (Schmittgen and Livak 2008). *∆*CT values were used for statistical analyses.

**Table 2.** Primer sequences.

Gene name	Forward sequence	Reverse sequence
SOCS3	5′‐TGCAGGAGAGCGGATTCTAC‐3′	5′‐TGACGCTCAACGTGAAGAAG‐3′
Pax 7	5′‐GCTACCAGTACAGCCAGTATG‐3′	5′‐GTCACTAAGCATGGGTAGATG‐3′
myogenin	5′‐CTACAGGCCTTGCTCAGCTC	5′‐AGATTGTGGGCGTCTGTAGG‐3′
GAPDH	5′‐TGCACCACCAACTGCTTAG	5′‐GGATGCAGGGATGATGTTC‐3′

### Proliferation phenotype experiment using human serum

To determine the effects of systemic factors on C2C12 myoblasts proliferation at a phenotypic level, passage 8 cells were seeded in cell‐treated 96‐well plates at a concentration of 1000 cells per well in 100 *μ*L GM. Plates were incubated at 37°C in 5% CO_2_ environment for 24 h to allow myoblasts to adhere and grow. GM was removed and cells were treated with 200 *μ*L of treatment media consisting of DMEM supplemented with 5% serum from children with CF or from healthy controls, with each time point represented (i.e., REST, EX, and REC) and 1% penicillin–streptomycin. Treated plates were incubated at 37°C in 5% CO_2_ for 1 h, after which serum was removed and replaced with GM. Plates were then incubated at 37°C in 5% CO_2_ for 2 days. After 2 days of incubation, plates were washed with 200 *μ*L of PBS, then treated with 2% PFA at a volume of 100 *μ*L, and incubated at room temperature for 30 min. Plates were then washed with 200 *μ*L of PBS, treated with 50 *μ*L of DAPI, and incubated for 10 min in the dark. Plates were washed with 200 *μ*L of PBS, air dried, and stored at 4°C until analysis. The number of nuclei was assessed using a Nikon Eclipse Ti (Nikon Instrument Inc. Melville, NY) at 10× magnification. Five random fields of view were captured from each well and the number of nuclei was determined using the NIS‐Element AR 3.2 64‐bit (Nikon Instrument Inc.) program. The sum of the nuclei from all five photographs was used to represent the total number of nuclei for each well.

### Plasma analysis

Given the possible implication of IL‐6's involvement in muscle proliferation (Toth et al. 2011) plasma samples were analyzed for IL‐6 using enzyme‐linked immunosorbent assays (ELISA) from R&D systems (Human IL‐6 Quantikine HS ELISA kits, Minneapolis, MN). All exercise concentrations were corrected for changes in plasma volume (Dill and Costill 1974).

### Statistical analyses

Statistical analyses were completed using SPSS version 17.0 (PASW Statistics version 17.0, SPSS Inc., Chicago, IL), unless otherwise stated. Data were tested for normality using Shapiro–Wilk test. All variables were normally distributed except for height percentile and IL‐6. To determine differences in participants' characteristics, an independent *T*‐test was used for normally distributed data and the Mann–Whitney *U‐*test was used for non‐normally distributed data. To determine differences between groups in protein expression, gene expression and proliferation phenotype and signaling two‐way repeated measure ANOVAs were performed. The two factors examined were group (CF, Control) × time (REST, EX, REC). To determine the effects of exercise, one‐way repeated measure ANOVA was preformed separately for children with CF and controls. Tukey's HSD post hoc analyses were performed to test for differences between means. All ANOVA analyses were completed using STATISTICA version 5.0 (StatSoft Inc., Tulsa, OK). To determine difference between groups in plasma IL‐6, the Mann–Whitney *U*‐test was used for each time point (i.e., REST, EX, and REC). To determine the effects of exercise on plasma IL‐6 concentration, Kruskal–Wallis *H* test with Wilcoxon post hoc was used. Values are expressed as mean ± SD unless stated otherwise. Significance was set at *P *≤**0.05.

## Results

### Protein expression

**Figure 1.**
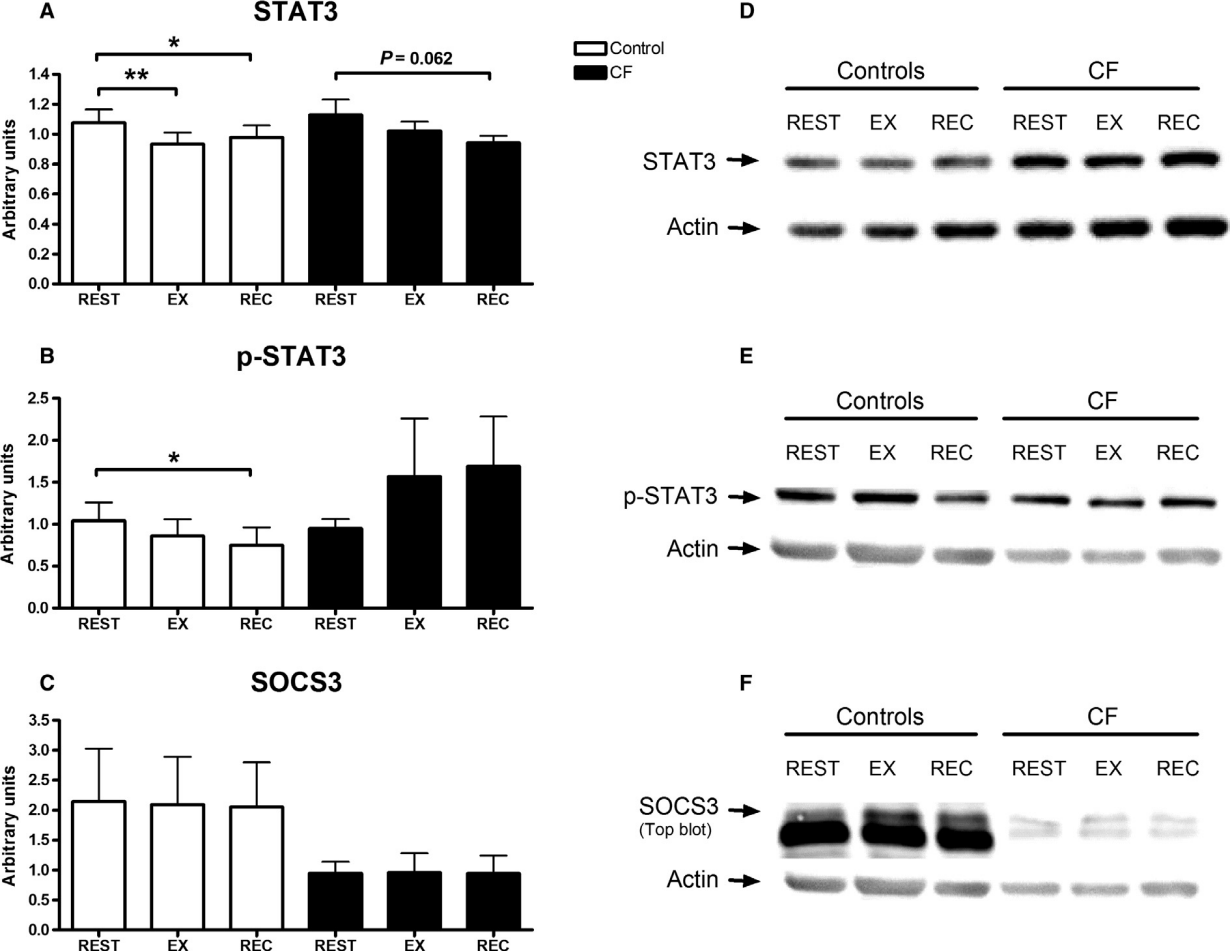
(A) STAT3, (B) STAT3 phosphorylation (p‐STAT3), and (C) SOCS3 expression in myoblasts treated with CF or healthy serum. (D) STAT3, (E) p‐STAT3, and (F) SOCS3 western blots. Myoblasts were treated for 1 h and harvested immediately after. Data are expressed in mean ± SEM normalized to actin. STAT3: *n *=**8 for each condition; p‐STAT3: control, *n *=**7, CF,* n *=**7; SOCS3: control, *n *=**7, CF,* n *=**8. There were no differences between groups. Significant difference from REST: *P <**0.05, **P <**0.001. A trend was observed with STAT3 in C2C12 myoblasts treated with CF serum (*P *=**0.062).

There was no significant difference in STAT3, p‐STAT3, or SOCS3 between C2C12 myoblasts treated with serum from children with CF or healthy controls (Fig. 1). There was no exercise effect observed with any of the protein markers in C2C12 myoblasts treated with serum from children with CF, although a trend toward a decrease in STAT3 at REC compared to REST was observed (*P *=**0.062). An exercise effect was observed for STAT3 and p‐STAT3 expression in C2C12 myoblasts treated with healthy serum; STAT3 decreased with EX (*P *<**0.01) and REC (*P *<**0.05) compared to REST. Expression of p‐STAT3 decreased in C1C12 myoblasts treated with healthy serum at REC (*P *<**0.05) compared with REST, while EX had no effect of p‐STAT3.

### mRNA expression

**Figure 2.**
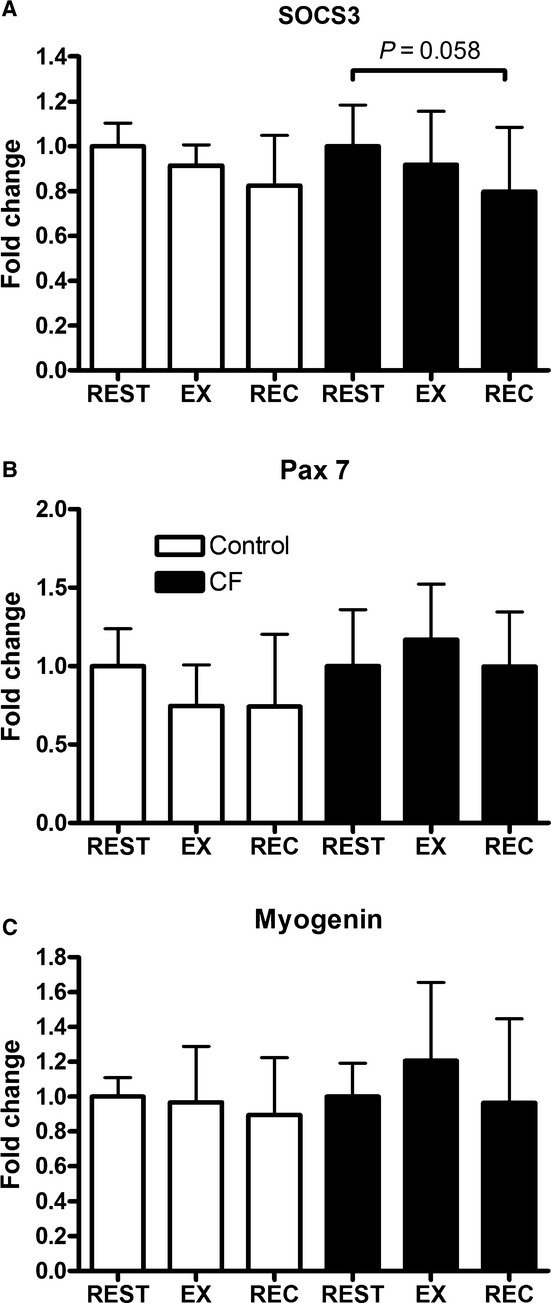
Gene expression expressed as fold change ± SD from REST for (A) SOCS3, (B) Pax7, and (C) myogenin in C2C12 myoblasts treated with CF serum or control serum. Myoblasts were treated for 1 h and harvested immediately after. C2C12 myoblasts were treated with serum from REST: before exercise, EX: after 60 min of cycling, REC: after 60 min of recovery. *n *=**3 for each condition. In C2C12 myoblasts treated with CF serum a trend (*P *=**0.058) toward reduced SOCS3 gene expression at REC from REST was observed.

Similar gene expression of SOCS3, Pax7, and myogenin were observed in C2C12 myoblasts treated with serum from children with CF or healthy controls (Fig. 2). In C2C12 myoblasts treated with serum from children with CF, a trend for an exercise effect for SOCS3 was observed (one‐way ANOVA, time effect, *P *=**0.065). In addition, a reduction in gene expression of SOCS3 at REC from REST (*P *=**0.058) (Fig. 2A) was observed. No exercise effect was observed in C2C12 myoblasts treated with serum from either children with CF or healthy controls for myogenin or Pax7.

### Proliferation

**Figure 3.**
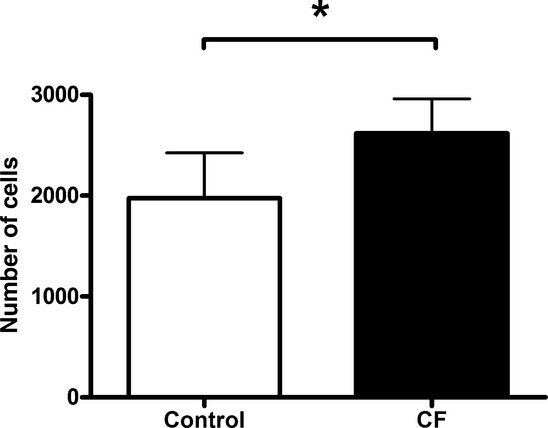
Myoblast proliferation. Average number of C2C12 cells treated with either CF or control serum. Data are expressed as mean ± SD. Wells were seeded with 1000 myoblasts in 100‐*μ*L growth media and allowed to adhere for 24 h. Myoblasts were then treated for 1 h with treatment media and allowed to proliferate in growth media for 2 days. *Significant difference between groups, *P *<**0.001.

**Figure 4.**
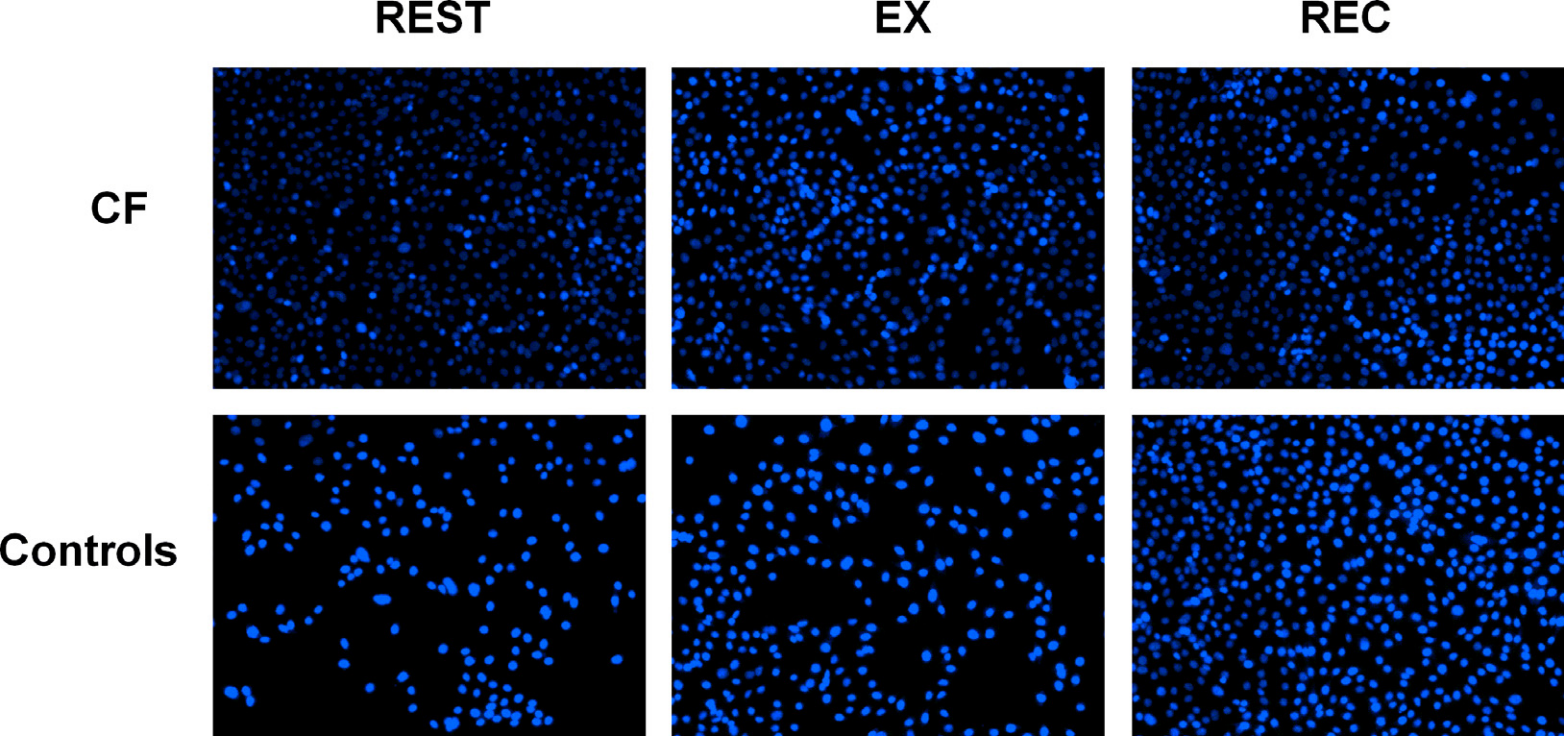
Photographs of myoblasts treated with serum from children with CF or healthy controls. Wells were seeded with 1000 myoblasts in 100‐*μ*L growth media and allowed to adhere for 24 h. Myoblasts were then treated for 1 h with treatment media and allowed to proliferate in growth media for 2 days. Myoblasts nuclei were stained with DAPI. REST: before exercise, EX: after 60 min of cycling, REC: after 60 min of recovery. *n *=**12 for each conditions.

**Figure 5.**
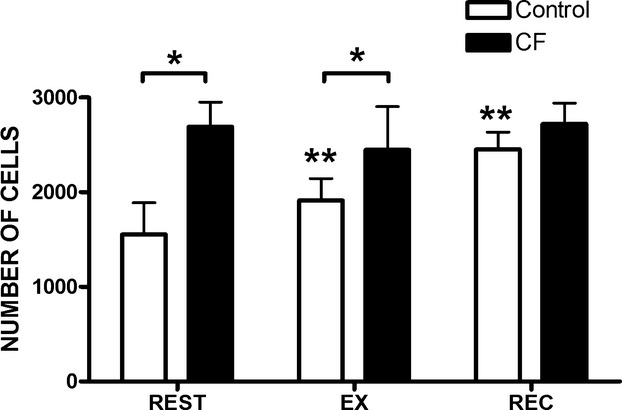
Effects of serum from children with CF and healthy controls on myoblast proliferation. Wells were seeded with 1000 myoblasts in 100‐*μ*L growth media and allowed to adhere for 24 h. Myoblasts were then treated with treatment media for 1 h and allowed to proliferate in growth media for 2 days. Mean ± SD number of nuclei. REST: before exercise, EX: after 60 min of cycling, REC: after 60 min of recovery. *n *=**12 for each conditions. *Significant difference between groups, *P *<**0.001. **Significant difference compared to REST,* P *<**0.01.

Proliferation of C2C12 myoblasts treated with serum from children with CF was greater than with healthy controls (main effect for group, *P *<**0.001) (Fig. 3) 2 days after treatment. C2C12 myoblasts treated with serum from children with CF showed greater proliferation at REST (*P *<**0.001) and at EX (*P *<**0.001) compared to healthy controls, but proliferation was similar at REC (Figs. 4–5). Exercise serum from children with CF had no effect on C2C12 proliferation. Exercise serum from healthy controls increased proliferation in C2C12 with values higher at EX (*P *<**0.001) and REC (*P *<**0.01) compared to REST.

### Plasma IL‐6

**Figure 6.**
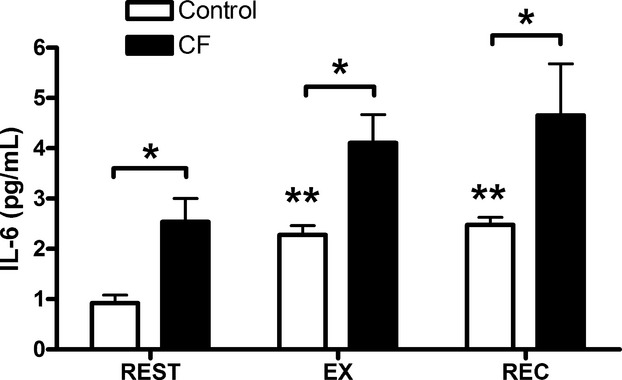
Effects of exercise on systemic IL‐6 in children with CF and controls. Data are expressed in mean ± SEM. REST: before exercise, EX: after 60 min of cycling, REC: after 60 min of recovery. *n *=**11 for each condition. *Significant difference between groups, *P *<**0.05. **Significant difference compared to REST,* P *<**0.01.

There was a significant difference in plasma IL‐6 between groups at each time point (Fig. 6). Exercise tended to increase IL‐6 in the children with CF, but this did not reach statistical significance (*P *=**0.066). Exercise increased IL‐6 in the healthy controls (*P *<**0.01) and remained elevated at REC (*P *<**0.01).

## Discussion

C2C12 myoblasts proliferated to a greater extent when treated with serum from children with CF compared to healthy control serum. One reason for this difference may be related to the higher concentration of IL‐6 observed in our CF group, as IL‐6 is known to induce myoblast proliferation via the JAK/STAT3 pathway (Toth et al. 2011). According to the literature, other systemic factors may be playing a role in myoblast proliferation. IL‐1 has the capacity to induce greater myoblast proliferation (Otis et al. 2014) and while we did not measure IL‐1 in our participants other studies have reported higher systemic levels in patients with CF (Greally et al. 1993). Therefore, although a higher concentration of IL‐6 in our CF serum may be responsible for inducing greater proliferation, other inflammatory mediators may also be involved.

While some mediators can induce greater myoblast proliferation, others can inhibit proliferation. Reduced oxidative stress has been shown to increased myoblast proliferation (Zaccagnini et al. 2007), suggesting that high oxidative stress would result in lower myoblast proliferation. In addition, NSAIDs (Mikkelsen et al. 1985) can inhibit myoblast proliferation and corticosteroid (te Pas et al. 2000) can reduce the rate of proliferation during the early stages of proliferation. We did not measure oxidative stress in our CF patients, however, increased oxidative stress is observed in CF patients compared to healthy subjects (Reid et al. 2007). In addition, six of our patients with CF were on NSAIDs and/or inhaled or nasal spray corticosteroids, and the effects of NSAIDs were not accounted for in our study. Despite the known effects of oxidative stress, NSAIDs and corticosteroids on reducing proliferation, our myoblasts treated with CF had greater proliferation. Pooling our samples may have diluted the concentrations of NSAIDs and corticosteroids and reduced the inhibitory effects on proliferation. Another scenario may be that the systemic factors that stimulate myoblast proliferation were much more potent than the inhibitory effects of oxidative stress, NSAIDs, and corticosteroids.

The effects of exercise on circulating inflammatory cytokines in children with and without CF (*n *=**12 for each group) from this study has been published elsewhere (Nguyen et al. 2012). While there were no exercise‐related changes in proliferation for myoblast treated with CF serum, differences were observed with healthy control serum. More specifically, the EX and REC serum from controls caused greater proliferation compared to REST serum. Given that IL‐6 concentrations were higher at these time points in healthy controls only, it is plausible that the proliferative property of IL‐6 may also explain increased C2C12 myoblasts proliferation in this group. Conversely, the lack of increase in myoblast proliferation with EX and REC serum from children with CF may be attributed to a ceiling effect. Systemic concentrations of IL‐6 were already much higher in the CF group at REST, and did not substantially increase at EX or REC. Additional experiments are required to determine the true role of IL‐6 in myoblast proliferation in this context. However, our data are novel as they highlight that serum from patients suffering from a systemic inflammatory disease can cause greater C2C12 myoblast proliferation. Furthermore, EX and REC serum from children with CF does not alter this proliferative response, while EX and REC serum from healthy controls enhances it.

In this study, C2C12 myoblasts proliferated to a similar extent when exposed to serum from children with CF (either with REST, EX, or REC) and REC serum from healthy controls. This suggests that in healthy children, the systemic environment created during the immediate recovery period following exercise induces similar effects on C2C12 myoblast proliferation as CF serum. An increase in myoblast proliferation is required for differentiation (Thomas et al. 2000); however, an increase in proliferation in C2C12 myoblasts when treated with an inflammatory stimulus results in reduced capacity to differentiate (Dogra et al. 2006). In children with CF, given their chronic inflammatory state (Tirakitsoontorn et al. 2001; Nguyen et al. 2012), it is conceivable that the increased proliferation observed with CF serum would be conducive to impaired differentiation leading to impaired muscle development. Indeed, young rats given chronic IL‐6 exposure experienced 13% reduced muscle growth (Bodell et al. 2009). However, it is difficult to speculate as to whether the observed increase in proliferation with recovery serum from healthy children is conducive to greater or impaired muscle development given that (1) the levels of IL‐6 observed in healthy children were significantly lower than the CF group at all time points including at the recovery time point, (2) the elevated inflammatory state is acute, and (3) the inflammatory state was induced by exercise and not by a chronic infection or disease. Thus, to further investigate the effects of increased proliferation observed on muscle development, examining the effects of myoblast differentiation after serum exposure would be the next logical step. Although in our study, mRNA markers of proliferation and differentiation (Pax7, SOCS3, and myogenin) were unaffected by exposure to serum, further work should measure differentiation phenotype (e.g., myoblast fusion index) of C2C12 myoblasts exposed to different systemic environments. On the basis of our data, we would expect C2C12 differentiation to decrease upon exposure to serum from children with CF.

Systemic factors from healthy children following exercise decreased protein signaling involved in C2C12 myoblast proliferation. Specifically, STAT3 and p‐STAT3 decreased in myoblasts treated with EX and REC serum from healthy controls, compared to REST serum. We expected these results to translate into less proliferation with myoblasts treated with EX and REC serum in our phenotype experiments. However, we observed the opposite effect with an increase in proliferation. These discordant findings may be due to timing, since protein samples were collected immediately following serum treatment, while the phenotype experiments were performed on samples collected 2 days after serum treatment. Our results may suggest that either (1) systemic factors promoted an acute reduction in proliferation signaling in C2C12 myoblasts, with a later increase in proliferation phenotype; or (2) the JAK/STAT3 pathway is not responsible for the increased proliferation phenotype observed.

Our study focused on comparing the effects of systemic factors in children with CF and healthy controls by using a common target tissue (C2C12 myoblasts). We acknowledge that the use of human serum on a mouse cell line may limit the application of our results since we investigated the effects of systemic factors from one species on another species' tissue. Because the use of human serum on tissue is a novel approach, we sought to insure the use of serum on a tissue was feasible and that differences would be apparent. We used the C2C12 cell line because relative to human primary or human cell lines C2C12 myoblasts are more proficient in growth, proliferation, and relatively inexpensive. Human cells are known to be difficult to grow, slow to proliferation, and expensive. Additionally, it is common to grow and proliferate C2C12 myoblasts using fetal bovine serum, and to differentiate using horse serum. Thus, using serum from a species other than from a mouse on C2C12 myoblasts is common practice. Given the feasibility of our study, we plan to pursue the use of a human cell line or human primary cells in the future. Furthermore, since the CFTR protein is expressed in skeletal muscle (Lamhonwah et al. 2010), future work should also include skeletal muscle from a CF model to provide clearer insight into the relative roles of the systemic environment and local muscle factors in skeletal muscle development in children with CF. Finally, we pooled our serum samples in order to conserve sample volume and to insure the completion of all other analyses set forth, since pediatric blood samples are difficult to obtain and standard ethical procedure inhibits the collection of blood to a certain amount. Pooling our samples is a limitation of this study.

## Conclusion

We took the novel approach of exposing C2C12 myoblasts to serum obtained from children with CF and healthy controls. We found that C2C12 myoblast proliferation was greater when treated with CF serum than control serum. In addition, proliferation did not differ between REST, EX, or REC CF serum, while an exercise effect was observed in healthy controls. Protein and mRNA markers of proliferation did not increase in C2C12 myoblasts treated with serum from children with CF or healthy controls. This work highlights the ability of systemic factors from patients with an inflammatory disease to alter aspects of skeletal muscle development.

## Acknowledgment

We would like to express our sincere appreciation to the participants and families involved for their dedication to the study. A special thanks to Valerie Carroll (Nurse Coordinator) and to the McMaster Pediatric CF Clinic for their assistance with patient recruitment.

## Conflict of Interest

None declared.
